# Spatiotemporal Comparisons Between Elite and High-Level 60 m Hurdlers

**DOI:** 10.3389/fpsyg.2019.02525

**Published:** 2019-11-15

**Authors:** Pablo González-Frutos, Santiago Veiga, Javier Mallo, Enrique Navarro

**Affiliations:** ^1^Faculty of Health Sciences, Francisco de Vitoria University, Madrid, Spain; ^2^Health and Human Performance Department, Technical University of Madrid, Madrid, Spain

**Keywords:** track and field, kinematics, performance analysis, competition, DLT algorithms

## Abstract

Despite the existence of literature on the athletics hurdles event, no previous studies have examined the kinematic behavior of athletes during the race. The aims of the present research were (1) to compare the spatiotemporal parameters of elite and high-level hurdlers (men and women) in the approach run, hurdles-unit and run-in phases and (2) to relate these parameters to the 60 m end race results. Split times, step lengths, step widths, step times, contact times and flight times were calculated for the 60 m hurdlers (*n* = 110) who participated in the 44th Spanish Indoor Championship and in the 12th IAAF World Indoor Championship. Both men and women elite-level hurdlers obtained shorter split times than high-level hurdlers in the approach run (δ 0.14 ± 0.01 and 0.18 ± 0.02 s, respectively), the hurdles-unit (δ 0.11 ± 0.01 and 0.13 ± 0.01 s, respectively) and the run-in (δ 0.10 ± 0.01 and 0.20 ± 0.02 s, respectively) race phases. Elite-level men athletes also presented lower step lengths in the approach run phase (δ 0.01 ± 0.00 m), greater take-off distances (δ 0.10 ± 0.03 m) and shorter landing distances (δ 0.17 ± 0.05 m) than high-level athletes, although elite-level women hurdlers only showed longer landing step length (δ 0.07 ± 0.02 m) than high-level athletes. Finally, in the run-in phase, elite-level hurdlers had longer step lengths than high-level hurdlers (men: δ 0.09 ± 0.03 m; women: δ 0.11 ± 0.03 m). Step times, contact times and flight times were also different between both levels of performance in most of the race phases. Correlational analysis with the race result showed large (*r* > 0.5), very large (*r* > 0.7), or nearly perfect (*r* > 0.9) relationships for most of the mentioned kinematic parameters. These results indicate that elite-level athletes were faster than high-level in the three phases of the 60 m hurdles event, specifically in some new spatiotemporal parameters (e.g. step length in the run-in phase) as well as others already studied. Accordingly, coaches and athletes should implement their training programs to have an impact on these key variables.

## Introduction

The International Association of Athletics Federations (IAAF) included the 60 m hurdles since the first World Indoor Championship (Indianapolis, USA) in 1987. The height and position of the hurdles from the starting line is the same in the 60 m as in the men 110 m and women 100 m hurdles events. The main difference between the events is the reduction of the number of hurdles from 10 to 5 from the 110–100 to the 60 m hurdles event. The positioning of the hurdles serves as a reference for the division of the event into the following phases (Brüggemann, 1990): approach run phase (from the starting line to the first hurdle), hurdle unit phase (race and clearance of the hurdles), and run-in phase (from the last hurdle to the finishing line). Additionally, the hurdle unit phase is subdivided into preparatory, hurdle, landing, and recovery steps (McDonald and Dapena, 1991).

The most common analysis of the hurdle’s races has been carried out through an evaluation of the hurdle split times (from touchdown to touchdown behind the hurdle), using video recordings with a fixed or panned video-camera both in 110 and 100 m (Muller and Hommel, 1997; Graubner and Nixdorf, 2011; Tsiokanos et al., 2017; Pollitt et al., 2018a,b) as of 60 m hurdles (Walker et al., 2019a,b). López del Amo et al. (2018) found that the reaction time and the approach run time were predictors of race performance, however, Tsiokanos et al. (2017) did not observed a significant relationship between reaction time and the race performance. Similarly, Tsiokanos et al. (2017) determined that the correlation between the intermediate times and final performance was decisive from the fifth hurdle onwards in the 110 m hurdles event (*r* = 0.77 – 0.98). The temporal analysis in the hurdle event is usually completed with the hurdle flight times, although there is a lack of relationship between hurdle clearance times and race performance according to Tsiokanos et al. (2017). Also, Pollitt et al. (2018a,b) have studied the contact and flight times during the run-in phase in the 110 and 100 m hurdles event.

Technical information of the hurdle events has been completed with two- and three-dimensional kinematic analyses of a single hurdle, which is usually selected between the second and sixthhurdle (Mann and Herman, 1985; Rash et al., 1990; McDonald and Dapena, 1991; Coh and Dolenec, 1996; Salo et al., 1997; McDonald, 2002; Coh, 2003; Park et al., 2011; Ryu and Chang, 2011; Coh and Iskra, 2012; Pollitt et al., 2018a,b). There is only one precedent (Ho et al., 2020) who have analyzed all ten hurdles of a 110 m hurdle event, measuring take-off (2.04 ± 0.07 m) and landing (1.47 ± 0.03 m) distances. Findings from these kinematical analyses suggest that efficient hurdle clearance technique is associated to the take-off contact time, take-off to landing point ratio in relation to the hurdle and to the hurdle flight time. In addition to the hurdle unit phase, the approach run phase (Rash et al., 1990; López del Amo et al., 2018; Walker et al., 2019a,b) has been subjected to kinematic analysis. To our knowledge, the run-in phase has been excluded from all the investigations carried so far, except for the research carried out by Pollitt et al. (2018a,b). The fact that most studies have been carried out in training situations, including a low sample of non-elite athletes, makes it difficult to obtain benchmark values representative of elite-level practitioners of the hurdle event performance.

Therefore, the aims of the present research were: (1) to compare the distance and time variables of elite-level and high-level hurdlers (men and women) in the approach run phase, hurdles phase and run-in phase, and (2) to relate these variables to the end race results. Our hypothesis is that elite-level hurdlers would be faster than high-level athletes in the three race phases, with a different step length pattern in some steps of the race. Additionally, we hypothesize that these differences in the step patterns will have a relationship with the end race result.

## Methods

All the races were filmed during the 60 m hurdle event of the 44th Spanish Indoor Championship and 12th IAAF World Indoor Championship (2008). The best performance of each participant men (*n* = 59) and women athlete (*n* = 51) from the heats, semifinal and final rounds were included in the study (Table 1). These performances were further subdivided into two groups (elite-level and high-level), according to the median of their official times achieved during the competition. All experimental procedures were carried out in accordance with the Declaration of Helsinki and were approved by the Ethics Committee of the Technical University of Madrid.

**Table 1:** Sample size (*n*), age, and end race result of the men and women athletes who participated in the 60 m hurdle races of the 44th Spanish Indoor and 12th IAAF World Indoor Championships.

Gender	Level	Age (years)	Race time (s)
Men (*n* = 59)	Elite-level (*n* = 30)	26.8 ± 3.6	7.71 ± 0.12 (7.46 – 7.93)
	High-level (*n* = 29)	22.6 ± 3.9	8.39 ± 0.28 (7.97 – 8.93)
Women (*n* = 51)	Elite-level (*n* = 27)	26.3 ± 3.3	8.14 ± 0.20 (7.80 – 8.46)
	High-level (*n* = 24)	22.9 ± 4.5	9.06 ± 0.32 (8.54 – 9.72)

**Figure 1:**
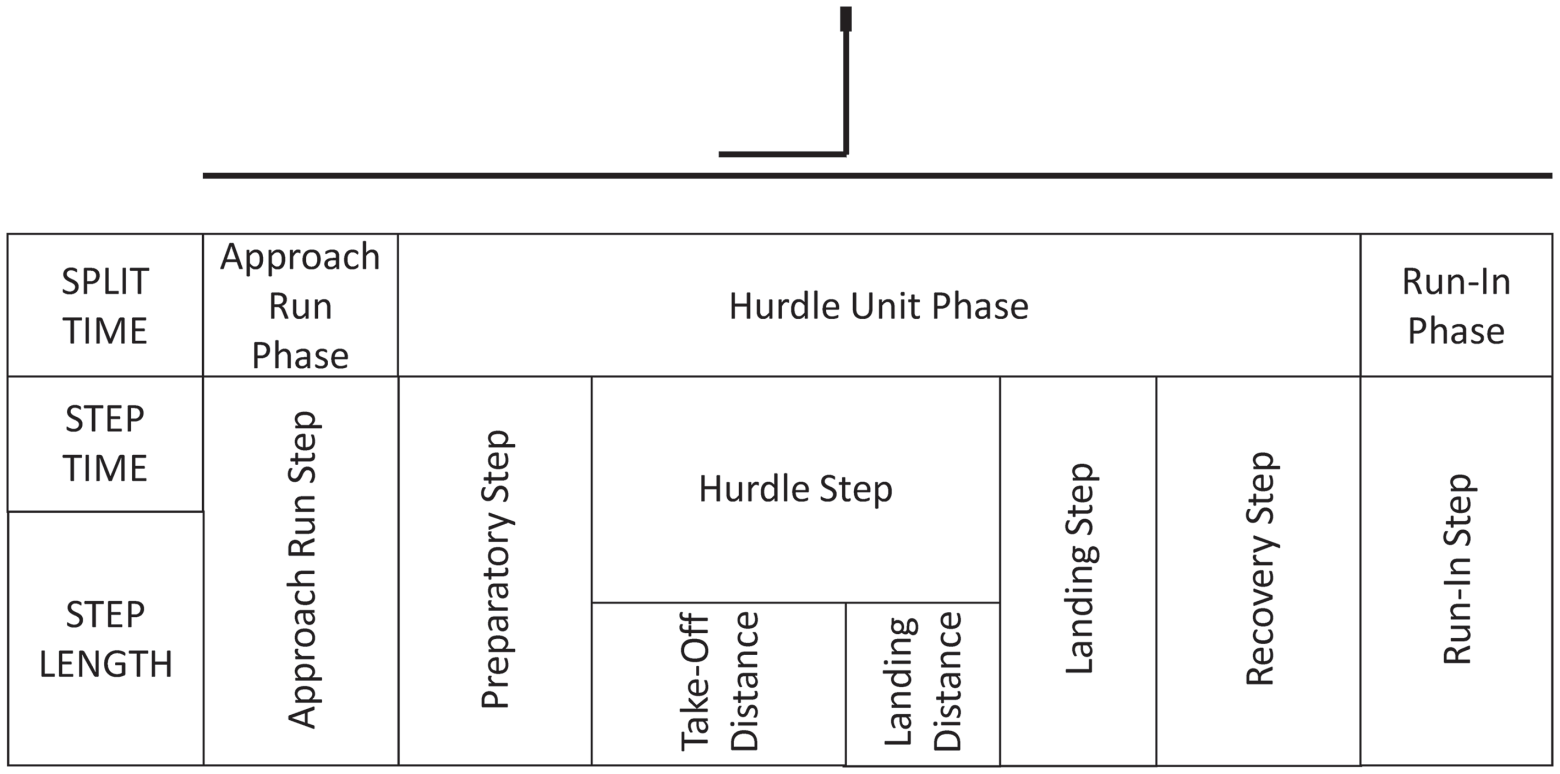
Race phases and Hurdle Unit Phase model (based on McDonald and Dapena, 1991).

The races were analyzed according to the following phases: approach run phase, hurdle unit phase, and run-in phase. For the split times, the approach run phase was calculated from the starting gun to the contact on the ground after the first hurdle, the hurdle unit phase was recorded as the mean of the times between each hurdle and the run-in phase included the time from the contact after the last hurdle to the final race time (Brüggemann, 1990). For the spatial analysis, the following model was used (McDonald and Dapena, 1991): (1) the approach run phase included the first eight steps before the first hurdle (the four men athletes who carried out seven steps in this phase were excluded from the analysis), (2) the hurdle unit phase (Figure 1) integrated the preparatory step, hurdle step (divided into take-off distance and landing distance), landing step and recovery step, and (3) the run-in phase which contained the steps between the last hurdle and the finish line.

**Figure 2:**
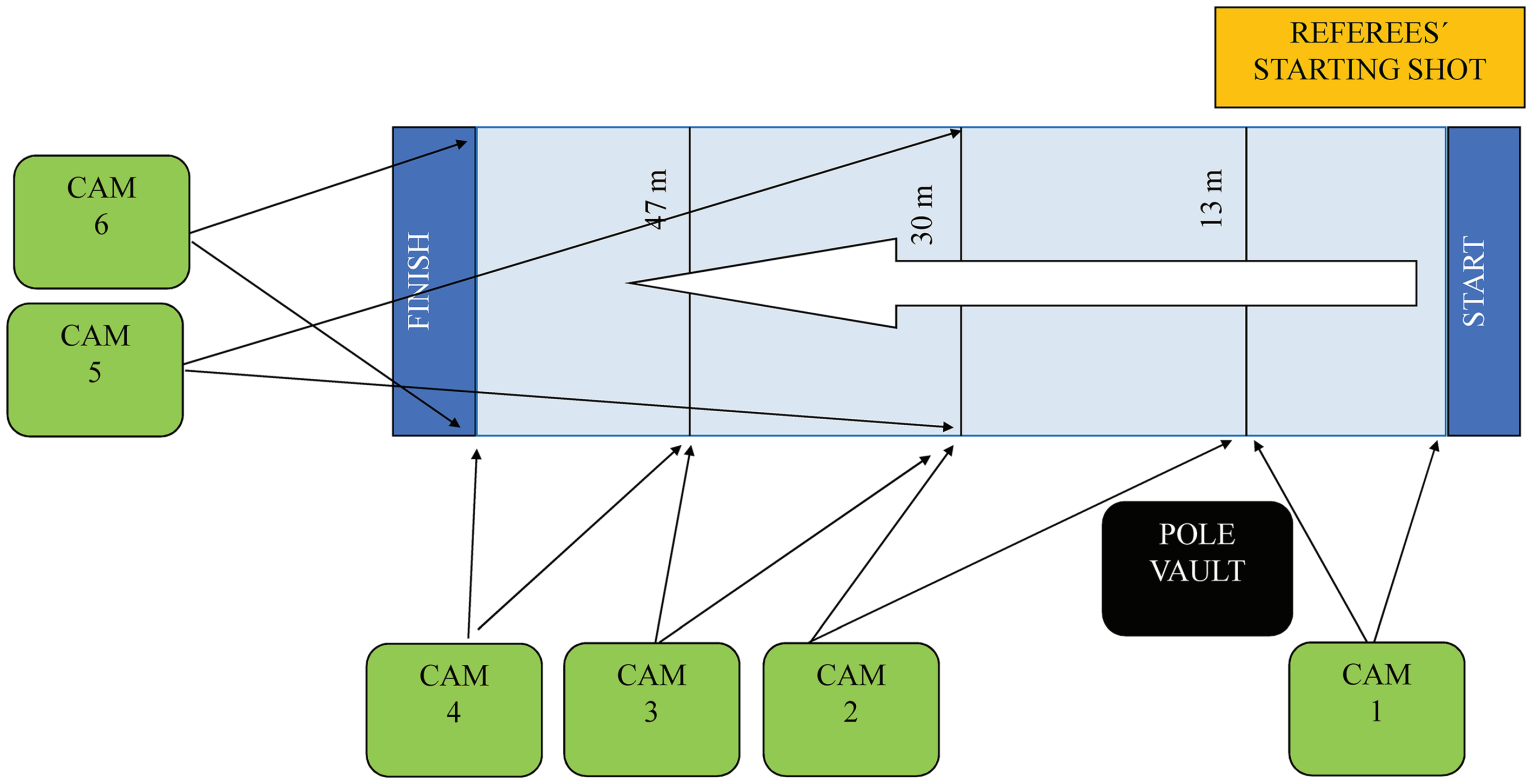
Camera setup position.

Six fixed video cameras JVC GY-DV300 (Japan Victor Company, Japan) located at the main stands and operating at 50 Hz (shutter speed: 1/1,000) were used to filmed the races, similarly to that previously described in other studies (McDonald and Dapena, 1991; Coh and Dolenec, 1996; Salo et al., 1997; Coh, 2003; Graubner and Nixdorf, 2011; Coh and Iskra, 2012). Camera 1 recorded the first 13 m; camera 2 from 13 to 30 m; camera 3 from 30 to 47 m, and camera 4 the last 13 m (47–60 m) of the race. Complementarily, and in order to avoid athletes’ visual occlusion, cameras 5 and 6 were located with a frontal view: camera 5 filming the first 30 m (including the referees’ starting gun) and camera 6 the last 30 m (Figure 2).

The athletes’ foot landing and take-off points were identified and manually digitized by an experimental observer from the race footage using Photo 23D software (Technical University of Madrid, Spain; Cala et al., 2009). Six control points, uniformly distributed in each camera view and represented by official line marks, were employed for calibration purposes and Direct Linear Transformation algorithms (Abdel-Aziz and Karara, 1971) were used to reconstruct the real coordinates (in meters) from the screen coordinates (in pixels). The measurements were validated and a Root Mean Square Error (Allard et al., 1995) lower than 0.04 m was determined for the step length and step width on the six cameras, in line with previous research on race analysis (Veiga et al., 2013). Intra-observer reliability by repeatedly digitizing (30 times) the same steps sequence in the eight competition lanes was 0.02 m in both axes.

Statistical analyses were performed using IBM SPSS statistics for Windows, version 22.0 (IBM Corp, Armonk, NY, United States). Split times, step lengths, step times, contact times, and flight times of the athletes were compared with a repeated measures analysis of variance according to the race phase (approach run phase, hurdle unit phase, preparatory step, hurdle step, take-off distance, landing distance, landing step, recovery step, and run-in phase), gender (men or women), and competitive level (elite-level or high-level). Planned repeated contrast tests between successive race phases were carried out. *Post hoc* tests were used to determine statistical effects (*p* < 0.05) between factors using Bonferroni corrections and were interpreted using effect sizes (partial *η*^2^) with 0.01, 0.06, and 0.14 threshold values for small, medium, and large effects (Cohen, 1992). Pearson correlation coefficients were used to relate all the spatiotemporal race parameters to the end race results, being 0.1, 0.3, 0.5, 0.7, and 0.9, the threshold values that represented small, moderate, large, very large, and nearly perfect correlations (Hopkins et al., 2009).

## Results

Split times during the competitive 60 m hurdles races (Table 2) had inter-level differences in all race phases, both for men (*F*_1.42_ = 6.30, *p* = 0.007, *η*^2^ = 0.09) and women (*F*_1.60_ = 9.98, *p* = 0.001, *η*^2^ = 0.17) athletes.

**Table 2:** Split times (s) of elite-level and high-level (men and women) hurdlers on the approach run, hurdle unit and run-in race phases during the 60 m event of the 44th Spanish Indoor and 12th IAAF World Indoor Championships.

		Approach run phase	Hurdle unit phase	Run-in phase
Men	Elite-level	2.64 ± 0.01^*^	1.04 ± 0.01^*^	0.90 ± 0.01^*^
	High-level	2.77 ± 0.01	1.15 ± 0.01	1.00 ± 0.01
Women	Elite-level	2.68 ± 0.01^*^	1.03 ± 0.01^*^	1.36 ± 0.01^*^
	High-level	2.86 ± 0.01	1.16 ± 0.01	1.55 ± 0.01

* *Inter-level statistical differences at a *p* level of 0.001*.

**Figure 3:**
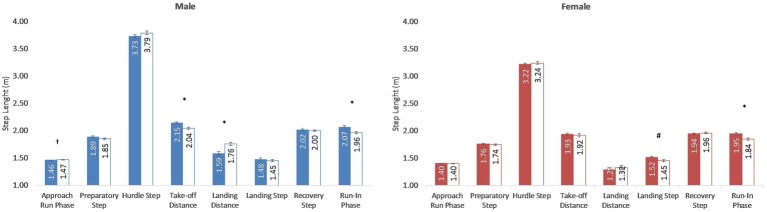
Mean step length (*m*) in the race phases of the elite-level (colored) and high-level (white) men and women athletes participating in the 60 m hurdler race of the 44th Spanish Indoor and 12th IAAF World Indoor Championships. Statistical inter-level differences: ^†^*p* < 0.05; ^#^*p* < 0.01; ^*^p < 0.001.

Figure 3 shows mean steps lengths for men athletes, with elite-level participants achieving a shorter step length than high-level athletes (δ 0.01 ± 0.00 m; *p* = 0.03) in the approach run phase and a longer step length in the run-in phase (δ 0.09 ± 0.02; *p* = 0.004). In the case of women athletes (Figure 3), the elite-level group had a greater length in the landing step than the high-level participants (1.52 ± 0.02 m vs. 1.45 ± 0.02 m, *p* = 0.009, respectively) and in the run-in phase (1.95 ± 0.02 m vs. 1.84 ± 0.02 m, *p* = 0.001, respectively). Specifically, during the hurdle step elite-level men athletes presented a greater take-off distance (elite-level: 2.15 ± 0.02 m, high-level: 2.04 ± 0.02 m; *p* = 0.001, *η*^2^ = 0.27) and a shorter landing distance (elite-level: 1.59 ± 0.03 m, high-level: 1.76 ± 0.03 m; *p* = 0.001, *η*^2^ = 0.27), whereas no significant inter-level differences were observed for women athletes both in the take-off and landing distances.

**Figure 4:**
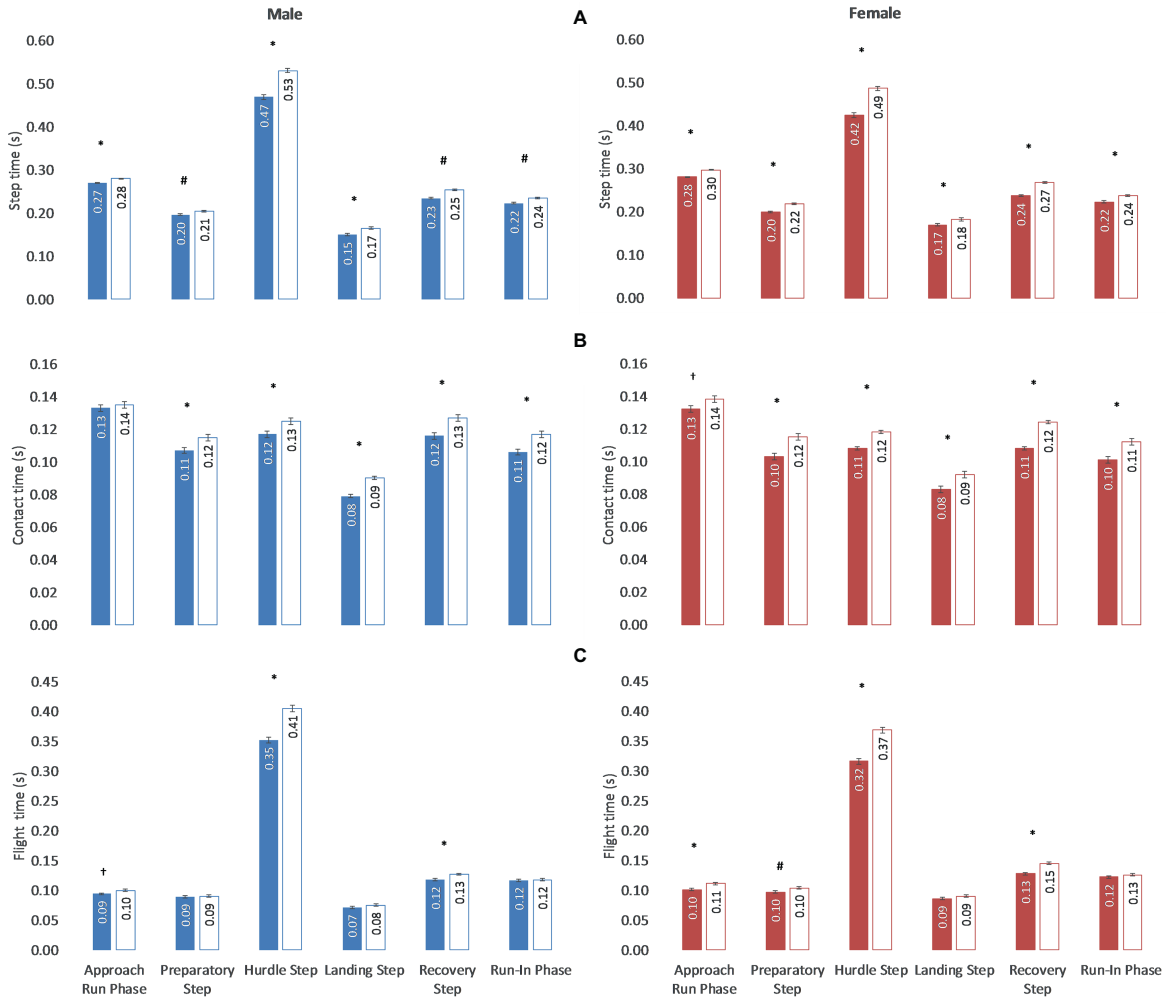
Step **(A)**, contact **(B)** and flight **(C)** times (*s*) in the race phases of the elite-level (colored) and high-level (white) athletes (men and women) participating in the 44th Spanish Indoor and 12th IAAF World Indoor Championships. Statistical inter-level differences: ^†^*p* < 0.05; ^#^*p* < 0.01; ^*^p < 0.001.

Step times (Figure 4A) had meaningful differences according to the level of competition in all phases both in men (*F*_2.30_ = 22.34, *p* = 0.001, *η*^2^ = 0.30) and women (*F*_2.56_ = 22.81, *p* = 0.001, *η*^2^ = 0.32) athletes. Contact times of elite-level men and women athletes (Figure 4B) were shorter (*p* = 0.05–0.000) than for high-level participants in all the phases of the race, except for the approach run in the men category. Flight times of men athletes (Figure 4C) had significant inter-level differences (*p* = 0.023) in the approach run phase while, in the hurdle unit phase, the elite-level athletes obtained a shorter flight time in the hurdle (elite-level: 0.35 ± 0.01 s, high-level: 0.41 ± 0.01 s, *p* = 0.001) and recovery (elite-level: 0.12 ± 0.00 s, high-level: 0.13 ± 0.00 s, *p* = 0.001) steps. In the case of women athletes, inter-level flight time differences were observed in the approach run phase (elite-level: 0.10 ± 0.00 s, high-level: 0.11 ± 0.00 s, *p* = 0.001) and in the preparatory (elite-level: 0.10 ± 0.00 s, high-level: 0.10 ± 0.00 s, *p* = 0.012), hurdle (elite-level: 0.32 ± 0.01 s, high-level: 0.37 ± 0.01 s, *p* = 0.001) and recovery (elite-level: 0.13 ± 0.00 s, high-level: 0.15 ± 0.00 s, *p* = 0.001) steps.

The correlation analysis with the final time had nearly perfect relationships (*r* > 0.90, *p* = 0.001) with the split times (Table 3) in all the race phases in both men and women events. In addition, step times (Table 4) had large (*r* > 0.05, *p* = 0.001) or very large (*r* > 0.7, *p* = 0.001) correlations in all the race phases for both men and women athletes, specifically during the hurdle step (men: *r* = 0.869, *p* = 0.001; women: *r* = 0.881, *p* = 0.001). Contact times and flight times had moderate to very large correlations in some race phases for men athletes, and large to very large correlations for women athletes. In particular, the flight time during the hurdle step had high correlations for both men (*r* = 0.815, *p* = 0.001) and women (*r* = 0.822, *p* = 0.001) participants. Regarding the step length, a negative correlation was found in the run-in phase in men (*r* = −0.494, *p* = 0.001) and women (*r* = −0.555, *p* = 0.001) groups. Complementarily, a negative correlation was observed in the preparatory step (*r* = −0.294, *p* = 0.038) and in the landing step (*r* = −0.285, *p* = 0.042) for women hurdlers, whereas the final time in the race had a significant correlation with the final time in the take-off distance (*r* = −0.439, *p* = 0.001) and landing distances (*r* = 0.471, *p* = 0.001) in men hurdlers.

**Table 3:** Relationships (*r*) between split times and the 60 m hurdlers race results during the 44th Spanish Indoor and 12th IAAF World Indoor Championships.

	Approach run phase	Hurdle unit phase	Run-in phase
Men	0.91^*^	0.99^*^	0.94^*^
Women	0.92^*^	0.99^*^	0.97^*^

* *p < 0.001*.

**Table 4:** Relationships (*r*) between spatiotemporal parameters and the 60 m hurdlers race results during the 44th Spanish Indoor and 12th IAAF World Indoor Championships.

Variable	Approach run phase	Preparatory step	Hurdle step	Take-off distance	Landing distance	Landing step	Recovery step	Run-in phase
**Men**								
Step length	0.15	−0.13	0.21	−0.44^*^	0.47^*^	−0.18	−0.25	−0.49^*^
Step time	0.76^*^	0.56^*^	0.87^*^			0.52^*^	0.72^*^	0.53^*^
Contact time	0.24	0.40^*^	0.41^*^			0.55^*^	0.62^*^	0.62^*^
Flight time	0.26	0.16	0.82^*^			0.15	0.46^*^	−0.01
**Women**								
Step length	0.05	−0.29^†^	0.11	−0.20	0.21	−0.29^†^	0.09	−0.56^*^
Step time	0.85^*^	0.69^*^	0.88^*^			0.61^*^	0.87^*^	0.63^*^
Contact time	0.28^†^	0.59^*^	0.65^*^			0.61^*^	0.82^*^	0.57^*^
Flight time	0.51^#^	0.37^#^	0.82^*^			0.27	0.65^*^	0.26

† *p < 0.05*;

# *p < 0.01*;

* *p < 0.001*.

## Discussion

The present research aimed to compare the spatiotemporal parameters of elite and high-level 60 m hurdlers on the race phases of the 44th Spanish Indoor and 12th IAAF World Indoor Championships. Results indicate that elite-level athletes were faster than high-level in the three phases of the 60 m hurdles event, specifically in some new spatiotemporal parameters (e.g. step length in the run-in phase) as well as others already studied. No previous studies had ever examined the spatiotemporal characteristics of competitive athletes on the race phases of the 60 m hurdlers event.

### Split Times

Elite-level athletes had shorter split times in all race phases than high-level athletes (men: δ 5–11%; women: δ 7–14%). These results suggest that elite-level participants do not only perform a faster approach run (as also reported by López del Amo et al., 2018) and hurdle unit phases, but are significantly faster during the run-in phase.

The split times in the approach run phase of elite-level hurdlers (Table 2) were slightly greater than those reported in previous studies carried out with this standard of athletes (Muller and Hommel, 1997; Graubner and Nixdorf, 2011; Tsiokanos et al., 2017; López del Amo et al., 2018; Pollitt et al., 2018a,b; Walker et al., 2019a,b). These differences could be explained by the greater sample size employed in the present research (30 men and 27 women athletes) in comparison to the seven or eight finalists studied during the above-mentioned studies. In fact, the mean split times of the eight best hurdlers of the present research had similar values (2.59 s men and 2.63 s women) to those reported elsewhere. For the high-level hurdlers, split times in the approach run phase (2.77 s) were also faster than the times recorded by Ho et al. (2020) in an experimental set-up (2.86 s), which highlights the competitive level of the group of athletes examined in the current investigation.

For the hurdle unit phase, split times of elite-level hurdlers (Table 2) were also similar to those recorded for the first five hurdles by Muller and Hommel (1997), Graubner and Nixdorf (2011), Tsiokanos et al. (2017), Pollitt et al. (2018a,b) and Walker et al. (2019a,b). In addition, the average of the eight best hurdlers in the present research (1.02 s men and 0.99 women) equaled the best result mentioned. For the high-level male hurdlers, split times in the hurdle unit phase (Table 2) was greater than those registered by Ho et al. (2020) which contradicts what was found in the approach run phase. Probably, changing environment between competition and training situation could explain part of these differences.

For the run-in phase, split times of elite men hurdlers had similar values (δ 0.01 s) to those reported by Walker et al. (2019a) for the seven finalists. In case of women, elite hurdlers had greater split times (δ 0.06 s) to those registered by Walker et al. (2019b) for the eight finalists in the 2018 World Indoor Championship.

### Step Length

Elite-level men hurdlers had smaller step length than high-level in the approach run phase (Figure 3), which allowed them to achieve greater take off distances in the first hurdle unit phase. These differences were not observed in the women races, probably due to differences in the hurdle height between men and women events (McDonald and Dapena, 1991; Salo et al., 1997). Indeed, there is a tendency in the last years between elite men hurdlers to perform seven steps in the approach run phase (probably to increase the take-off distance in the first hurdle), although this does not seem to be accompanied by faster times (Walker et al., 2019a,b).

In the hurdle step, step length values (Figure 3) Were in line to data from the last World Championships (Pollitt et al., 2018a,b), although greater than what has been reported for male athletes (Coh, 2003; López del Amo et al., 2018) The in-depth analysis of the hurdle step length indicated that elite-level men hurdlers obtained longer take-off (δ 0.10 m) and shorter landing (δ 0.16 m) distances than those athletes of lower level. This confirms the importance of the take-off and landing distances on performance (Table 4), as previously indicated by Coh and Iskra (2012). Values reported in the literature (McDonald and Dapena, 1991; Coh and Iskra, 2012; Pollitt et al., 2018a) showed similar take-off distances (from 2.04 to 2.31 m) but shorter landing distances (from 1.32 to 1.58 m). Unlike men, women races did not show inter-level differences either in the take-off or in the landing distances (Figure 3). This could indicate that the women 60 m hurdles event might have a lower technical component than men races due to the lower height of the hurdles (McDonald and Dapena, 1991; Salo et al., 1997). Men hurdlers should aim to maximize their take-off distance to have an impact on the trajectory of the CM above the hurdle and, therefore, to minimize the landing distance.

In the remaining steps of the hurdle unit phase, the step lengths for men (Figure 3) coincided with those provided by McDonald and Dapena (1991) and Coh (2003). In the case of women, the step lengths and the landing distance (Figure 3) were shorter than those reported by McDonald and Dapena (1991), Coh and Dolenec (1996), and Pollitt et al. (2018a,b), whereas the recovery step distance was longer. For the landing step length, there were inter-level differences in the women athletes (δ 0.07 m), and this parameter showed moderate correlation with the race results (Table 4). Therefore, in the case of women, this landing step could represent a key aspect of the hurdle unit phase where additional improvements could be obtained probably related to strength gains. Finally, in the run-in phase, both elite-level men and women athletes had a longer step length than high-level athletes (2.05 and 1.95 m, respectively), as previously reported in 100 m sprinters (δ 0.12 m; Ito et al., 2006). In addition, correlations between step length and the race result in men (*r* = −0.49; *p* = 0.001) and women (*r* = −0.56; *p* = 0.001; Table 2) suggest that the best hurdlers are also better sprinters, as a high correlation between step length and sprint running velocity has been previously reported for men and women athletes (men: *r* = 0.86; *p* < 0.01; women: *r* = 0.74; *p* < 0.01; Ito et al., 1998).

### Step Time

Step times had differences in all race phases between the two levels of performance, both in men and women (Figure 4), with large and very large correlations with the race result. These data are in line with previous findings in women sprinters (*r* = 0.77; *p* < 0.01; Ito et al., 1998) and suggest the importance of the stride frequency on elite training programs. Indeed, a common practice between coaches (Iskra, 1995) is to train with reduced distances between hurdles (compared to official) to stress on step frequency. For the contact times, unlike men, elite-level women obtained shorter contact times in the approach run. However, in the rest of the race phases, elite-level athletes presented shorter contact times than those of high-level athletes, both men and women, indicating the importance of this parameter not only on the hurdle (Coh and Iskra, 2012) but also on the remaining race steps. The values for the hurdle-unit phase were similar than those presented by McDonald and Dapena (1991) during official competition, but shorter than those reported by Coh and Iskra (2012) during training situation. Because of this, coaches, in addition to developing short contacts (<0.13 s), should be careful when comparing training and competition values. Light times of elite-level athletes (men and women) in the approach run phase had small differences from those of high-level, being the greatest differences observed in the hurdle (δ 0.06 s men; δ 0.05 s women) and the recovery steps. Since it is customary for coaches to control the flight time at the hurdle step, it would be also recommended to register it also at the recovery step and the departure phase. In general, the registered values were similar to those observed in other championships (McDonald and Dapena, 1991; Muller and Hommel, 1997; Graubner and Nixdorf, 2011; Tsiokanos et al., 2017) or lower than those registered in by Rash et al. (1990), Salo et al. (1997), Coh (2003), and Ho et al. (2020). That is why the values in the present research (despite the low sampling rate of 50 Hz) seem to be representative of the competitive reality.

These results indicate that elite-level hurdlers achieved shorter step times differently in each race phase. In the approach run phase they obtained shorter flight times, whereas in the run-in phase they presented shorter contact times. Probably, high levels of inter-limb coordination (approach run phase) or reactive force (run-in phase) could be of a practical importance here. In the hurdle unit phase, on the other hand, a basic modification of the technical training between coaches, together with the inter hurdle distances, is to train with reduced height of hurdles (compared to official) to stress a shorter flight time over the hurdle and shorter contact times in the hurdle unit phase (Iskra, 1995).

### Correlation Analysis

From a global point of view, the parameters that obtained a nearly perfect correlation with the 60 m hurdle race results were the split times for the hurdle unit phase, run-in phase and approach run phase (in this order), both for men and women athletes. Compared to the 110 and 100 m hurdle events (Tsiokanos et al., 2017), correlations of the approach run and run-in split times with final result were greater in the present study. Therefore, it seems important to work on the three race phases to be competitive internationally. The correlation of the mean split time in the hurdle unit phase (*r* = 0.99) were greater than those obtained for the individual split times between 6th (*r* = 0.84 – 0.82) and 10th (*r* = 0.97 – 0.98) hurdles by Muller and Hommel (1997) and Tsiokanos et al. (2017). This would indicate that the mean split time in the hurdle unit phase could be a good performance indicator parameter for athletes and coaches. Finally, in the hurdle unit phase, large correlations with final results were also observed for recovery step time, hurdle step time and hurdle flight time (men and women). This is in line with coaches usually employing hurdle exercises performed with trail leg or lead leg (Iskra, 1995).

### Practical Application

Based on some of the spatiotemporal parameters presented in the present research, there are some performance indicators common to men and women: a greater step length in the run-in phase and a shorter step, contact and flight times in selected race phases. On the other hand, there are some specific variables important for each gender: a greater take-off distance (preceded by a shorter step length in the approach run phase) and a shorter landing distance in the case of men and a greater landing step and a shorter flight times at the preparatory step for women. Indeed, it could be stated that men seem to present a greater technical requirement on the hurdle unit phase, whereas women present inter-level differences before and after the hurdle phase. Also, from a global point of view, it seems necessary that hurdlers become also good sprinters and that they improve performance in all the race phases as, in the 60 m hurdle event (unlike the 110 or 100 m hurdle races), the three race phases performance is highly correlated to the final result.

## Conclusion

Elite-level male hurdlers on 60 m events performed shorter step times, with shorter contact times and shorter step lengths on the approach to the first hurdle, which allowed them to achieve greater take-off and shorter landing distances. Conversely, on the run-in phase, male and female athletes performed greater step lengths (moderate and large correlations with race result) with shorter contact times indicating a better ability to apply impulse during contact. Split times of the hurdle unit phase, run-in phase and approach run phase (in this order) had the greatest correlation with the race result. The hurdle flight time and the step times along the race were also largely correlated with race result. These results indicate that elite-level athletes were faster than high-level in the three phases of the 60 m hurdles event, specifically in some new spatiotemporal parameters (e.g. step length in the run-in phase) as well as others already studied. Accordingly, coaches and athletes should implement their training programs to have an impact on these key variables.

## Data Availability Statement

The datasets generated for this study are available on request to the corresponding author.

## Ethics Statement

The studies involving human participants were reviewed and approved by Ethics Committee of the Technical University of Madrid. Written informed consent for participation was not required for this study in accordance with the national legislation and the institutional requirements.

## Author Contributions

PG-F, SV, JM, and EN contributed to conception and design of the study and organized the database. SV and PG-F performed the statistical analysis and wrote the first draft of the manuscript. All authors contributed to manuscript revision, read and approved the submitted version.

### Conflict of Interest

The authors declare that the research was conducted in the absence of any commercial or financial relationships that could be construed as a potential conflict of interest.
